# Efficacy of haemoadsorption combined with continuous renal replacement therapy in patients with rhabdomyolysis and acute kidney injury: a retrospective study

**DOI:** 10.1093/ckj/sfae406

**Published:** 2024-12-17

**Authors:** Xiaochun Zhou, Yingying Yang, Peiyun Li, Fang Wang, Ling Zhang, Ping Fu

**Affiliations:** Department of Nephrology, Kidney Research Institute, West China Hospital of Sichuan University, Chengdu, China; Department of Nephrology, Guanghan Hospital of Traditional Chinese Medicine, Deyang, China; Department of Nephrology, Kidney Research Institute, West China Hospital of Sichuan University, Chengdu, China; Department of Nephrology, Kidney Research Institute, West China Hospital of Sichuan University, Chengdu, China; Department of Nephrology, Kidney Research Institute, West China Hospital of Sichuan University, Chengdu, China; Department of Nephrology, Kidney Research Institute, West China Hospital of Sichuan University, Chengdu, China; Department of Nephrology, Kidney Research Institute, West China Hospital of Sichuan University, Chengdu, China

**Keywords:** acute kidney injury, continuous renal replacement therapy, haemoadsorption, mortality, rhabdomyolysis

## Abstract

**Background:**

Clearance of circulating myoglobin is crucial to prevent further damage in patients with rhabdomyolysis (RM) and acute kidney injury (AKI). The objective of the present study was to evaluate the efficacy and safety of haemoadsorption (HA) combined with continuous renal replacement therapy (CRRT) in critically ill patients with RM and AKI.

**Methods:**

Patients with RM and AKI who received CRRT + HA or CRRT with concomitant creatine kinase (CK) >10 000 IU/l in our intensive care unit (ICU) between May 2021 and December 2023 were retrospectively included. The primary outcome was 90-day mortality; secondary outcomes were kidney function recovery and CK decline rate. Adverse events were also evaluated, including hypotension, circuit clotting, albumin leakage and blood loss. Propensity score matching and Cox retrospective analysis were performed.

**Results:**

A total of 111 RM patients with AKI were ultimately included. The ICU and in-hospital mortality were significantly lower in the CRRT + HA group compared with the CRRT group (ICU mortality: 18% versus 42%, *P* = .025; in-hospital mortality: 21% versus 42%, *P* = .048). However, the CRRT + HA group only showed a non-significant reduction in 90-day mortality compared with the CRRT group (47% versus 68%, *P* = .063). After treatment for 90 days, the number of patients with kidney function recovery was not significantly different between the CRRT + HA and CRRT groups (95% versus 84%, *P* = .639). Moreover, the incidence of hypotension and circuit clotting events did not increase during CRRT + HA treatment. In addition, the CRRT + HA group also appeared to have a higher rate of CK reduction and reduction of CK than the CRRT group at 24 and 48 hours after the initiation of CRRT. A multivariate Cox regression model demonstrated that CRRT + HA {hazard ratio [HR] 0.477 [95% confidence interval (CI) 0.234–0.972], *P* = .042}, mean arterial blood pressure [per 1 mmHg; HR 0.967 (95% CI 0.943–0.992), *P* = .009] and CRRT treatment duration [per 1 h; HR 0.995 (95% CI 0.992–0.998), *P* = .002] played a favourably important role in the survival prognosis of RM and AKI patients. In contrast, serum phosphate before RRT [per 1 mmol/l; HR 1.531 (95% CI 1.113–2.106), *P* = .009] and McMahon score [per 1 score; HR 1.15 (95% CI 1.006–1.313), *P* = .04] were independent risk factors for 90-day mortality.

**Conclusions:**

CRRT combined with HA therapy reduced ICU and in-hospital mortality in patients with RM and AKI and also had a cleansing effect on creatine kinase without significant adverse events.

## INTRODUCTION

Rhabdomyolysis (RM) is a complex clinical syndrome caused by various factors including trauma, ischaemia, compression, overexercise, convulsions, heat exposure, infections and drugs. These factors result in the decomposition and necrosis of skeletal muscle tissues, triggering the release of cellular contents such as myoglobin, creatine kinase (CK) and electrolytes into the circulation [1–5]. Clinical manifestations of RM include electrolyte disturbances (hyperkalaemia, hyperphosphataemia and hypocalcaemia), muscle weakness or pain, swelling and dark tea-coloured urine [1, 6]. However, it is noteworthy that ≈50% of patients may not present with typical clinical symptoms, leading to missed diagnosis and late complications [1, 6]. Acute kidney injury (AKI) is the most common late complication of RM [7–9]. It is estimated that ≈13–50% of RM patients develop AKI, with 26% requiring renal replacement therapy (RRT), and mortality rates ranging from 10% to 50% [4, 10, 11]. Given the severity of the epidemiological situation, our primary focus is on how to treat it quickly and effectively.

Myoglobin, with a molecular weight of 17 kDa, is a primary causative agent in AKI. Usually myoglobin reaches its maximum concentration in the blood within 12 h after muscle injury. The kidneys can eliminate half of the myoglobin in ≈3 h when kidney function is not limited and muscle injury is not sustained [12]. However, if a patient develops AKI, the half-life of myoglobin will be significantly prolonged. This prolonged presence of myoglobin inflicts renal damage by promoting vasoconstriction, obstructing renal tubules, causing oxidative stress and lipid peroxidation and inducing inflammation [1, 11, 13–17]. Fluid resuscitation and diuresis are strategies aimed at accelerating myoglobin excretion, but their effectiveness becomes limited when kidney function is impaired. Therefore, RRT stands as the only treatment available in such conditions [18]. Intermittent haemodialysis (IHD) can be used to quickly and effectively correct life-threatening electrolyte abnormalities [11]. However, in patients with severe RM, IHD may lead to rebound hyperkalaemia and acidosis [19]. Moreover, myoglobin is a medium molecular weight solute that is unlikely to be removed by diffusion-based conventional haemodialysis (HD). Continuous RRT (CRRT) modalities, such as continuous venovenous haemofiltration (CVVH) or continuous venovenous haemodialysis filtration (CVVHDF), have the advantage of avoiding rebound from the internal environment and provide better removal of higher molecular weight solutes by convection rather than diffusion. However, studies on the efficiency of myoglobin removal have shown that myoglobin screening coefficients for CVVH using AV600 filters (Fresenius, Bad Homburg vor der Höhe, Germany) with a surface area of 1.35 m^2^ ranged from 0.11 to 0.28 under ultrafiltration conditions at 3 l/h [20]. In CVVHDF with 1 l/h ultrafiltration using AN69ST membranes (Gambro Renal Products, Lakewood, CO, USA), the screening factor for myoglobin was 0.21 [21]. The screening coefficient for myoglobin was <0.4 even with high-flux membranes [22]. It can be seen that CRRT can remove myoglobin, but the removal efficiency is limited, and there are differences in the removal effects of filters with different pore sizes and fluxes as well as filters with different therapeutic doses. There is still a need to continue to explore more effective myoglobin removal protocols.

To enhance myoglobin clearance, researchers have recently proposed a therapeutic approach combining haemoadsorption (HA) with CRRT. HA facilitates the removal of solutes with molecular weights <60 kDa, while the use of a new adsorbent with a higher surface area:volume ratio ensures slower saturation [23–25]. Several case reports have indicated that combining HA with CRRT can rapidly eliminate myoglobin, with a reduction between 41% and 50% and saturation of the cartridge at 12 h [26, 27]. However, comprehensive studies are lacking on the outcome events, safety events and myoglobin clearance efficiency of CRRT + HA in patients with RM and AKI.

## MATERIALS AND METHODS

### Patients

We conducted a retrospective cohort study in the intensive care unit (ICU) of the West China Hospital of Sichuan University from May 2021 to December 2023. First, we retrieved patients who met the diagnostic criteria for RM (CK >1000 IU/l or CK level five times higher than the standard value [4]) from the electronic medical records system. Patients who met the following criteria were eligible to participate: CK ≥10 000 U/l; patients admitted to the ICU; patients with AKI by the Kidney Disease: Improving Global Outcomes (KDIGO) definition [28]; and CRRT > 24 h and/or HA >8 h. Exclusion criteria were as follows: chronic kidney disease (CKD) diagnosed before admission; myositis; receiving extracorporeal membrane oxygenation therapy; patients receiving other blood purification regimens other than CVVHDF and CVVHDF + HA380; and a significant decrease in CK levels at the start of treatment.

This study was approved by the ethics committee of the West China Hospital of Sichuan University (20232061). Written informed consent for participation was not required for this study, in accordance with national legislation and institutional requirements. All patient data were kept confidential.

### Data collection

Data were retrieved from the electronic medical records system for patients started CRRT and/or HA. Demographic data included age, sex, comorbidities (hypertension, diabetes, neoplasms and chronic pulmonary disease) and discharge diagnosis. The severity of the disease was evaluated by the Acute Physiology and Chronic Health Evaluation II (APACHE II) score, the Sequential Organ Failure Assessment (SOFA) score and the McMahon score. The general condition of patients included pulse rate, mean arterial pressure (MAP), oxygen saturation at the initiation of therapy, use of vasoactive drugs and use of mechanical ventilation. Laboratory data included leucocytes, haemoglobin, platelets, prothrombin time (PT), fibrinogen (FIB), calcium, phosphate, potassium, kidney function markers [blood urea nitrogen (BUN), serum creatinine (SCr), estimated glomerular filtration rate (eGFR)], liver function markers [alanine aminotransferase (ALT), aspartate aminotransferase (AST), lactate dehydrogenase (LDH), albumin, globulin, total bilirubin and CK]. AKI was diagnosed and graded according to the 2012 KDIGO AKI guidelines [28]. Treatment included the start and stop time of CRRT and/or HA, treatment duration, anticoagulation modalities prescribed, number of HA treatments and blood transfusions. Patients were assigned to either the CRRT + HA or CRRT groups according to the type of blood purification therapy they received. The outcome data included recovery of kidney function, safety events (hypotension, coagulation events, albumin loss, haemoglobin depletion and platelet depletion), mortality, ICU stay duration and hospitalization. Survival time was defined as the duration from onset to death; if the survival time was >90 days, it was recorded as 90 days.

### CRRT protocol

CRRT was performed using the CVVHDF method with AN69ST-150 (Gambro Renal Products) haemofilters through a Prismaflex CRRT system (Baxter, Deerfield, IL, USA). Blood flow rates were maintained between 150 and 200 ml/min, and haemofilters were changed every 24 h to prevent premature clotting. The therapeutic dosage was 20–25 ml/kg/h. Bicarbonate replacement solution (Chengdu Qingshan Likang Pharmaceutical, Chengdu, China) was administered through a post-dilution method with replacement fluid/dialysate 1:1 (v:v). The appropriate anticoagulation mode was determined based on KDIGO recommendations.

The HA device was a disposable haemoadsorption device (HA380; Jafron, Zhuhai, China). Its adsorbent material is a secondary cross-linked polystyrene neutral macroporous adsorbent resin with a filling volume of 380 ml and an adsorption surface area of ≈54 000–64 000 m^2^, which can remove solutes with molecular weights of 5–60 kDa with relative specificity. In our study, the HA380 was connected in series after the filter, and before use it underwent a pretreatment procedure involving soaking in heparin for 30 min, followed by drainage with saline solution. Treatment sessions typically last 8–10 h. The frequency of HA therapy was tailored to individual patients and determined based on ongoing monitoring of their CK levels, myoglobin levels and the patient's condition.

### Definitions

Full recovery from AKI is achieved when patients no longer meet the AKI criteria after 90 days of follow-up [29]. Partial recovery of kidney function is defined as SCr not returning to normal levels and/or persistent proteinuria and haematuria after 90 days of follow-up, but without the need for HD [30]. Dependence on dialysis is defined as the need for HD after 90 days of follow-up [31]. The polyuria was defines as urine volume >2500 ml/day, without diuretics [20]. A significant decrease in CK levels was defined as a decrease in CK from its peak to <10 000 IU/l.

### Statistical analysis

Categorical variables were described as frequencies and percentages. Normally distributed continuous variables were expressed as mean ± standard deviation (SD), while continuous variables with non-normal distribution were presented as the interquartile range (IQR; the 25th–75th percentile). The baseline data were evaluated using statistical tests appropriate for their distribution, including the Student's *t*-test, Mann–Whitney U test, χ^2^ test or Fisher's exact test. Cox multivariable regression was performed to explore the relationships between variables and 90-day mortality by calculating hazard ratios (HRs) and 95% confidence intervals (CIs). Variables with *P*-values <.05 in the univariate analysis or those deemed as clinically significant or reported as meaningful in previous literature were included in the multivariate Cox proportional hazards regression model. The Kaplan–Meier curve was used to describe the survival probability of patients over time and intergroup comparisons were performed using the logrank test. To reduce the impact of baseline differences in demographic and clinical characteristics on patient outcomes, we employed the 1:1 propensity score matching (PSM) method to pair patients from the CRRT group with those from the CRRT + HA group. The matching ratio was 1, while a calliper value of 0.02. All statistics were calculated using SPSS Statistics version 27 (IBM, Armonk, NY, USA). For each analysis, two-sided *P*-values <.05 were considered statistically significant. The Kaplan–Meier survival curves were plotted using GraphPad Prism 10 XML (GraphPad Software, Boston, MA, USA).

## RESULTS

### Patient characteristics

**Figure 1:**
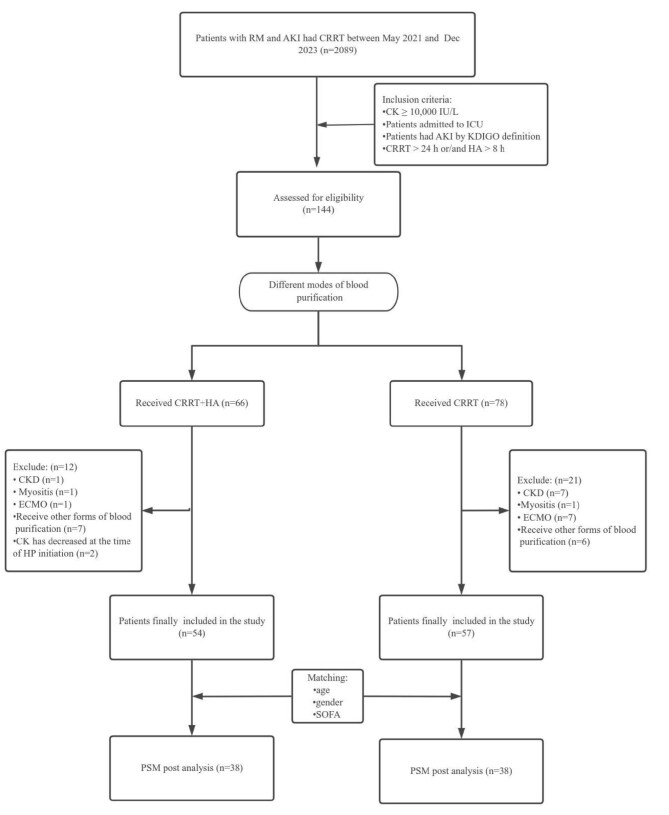
Flowchart of patient inclusion. CRRT >24 h and/or HA >8 h means that in the CRRT group patients received CRRT treatment for >24 h and in the CRRT + HA group patients received CRRT treatment for >24 h and HA therapy for >8 h.

From May 2021 to December 2023, a total of 2089 patients had a diagnosis of RM and received CRRT. After selection, 111 patients were enrolled in the final analysis, of whom 54 received CRRT + HA and 57 received CRRT. To address baseline imbalances, further PSM was performed considering gender, age and SOFA score. After PSM, each group consisted of 38 patients, ensuring comparability in baseline characteristics (Fig. 1).

Demographic characteristics and laboratory indexes are listed in Table 1. Before matching in the CRRT group and CRRT + HA group, trauma (12% versus 20%), sepsis (23% versus 17%) and pancreatitis (32% versus 26%) were identified as the most common causes of RM. Other causes included seizures (4% versus 6%), vascular ischaemia (10% versus 10%), bee stings (2% versus 2%), drugs (4% versus 4%), heat stroke (2% versus 5%), crush syndrome (2% versus 2%), myocardial infarction (5% versus 2%) and burn injury (4% versus 6%).

**Table 1:** Characteristics of the study population before and after PSM.

	Unmatched			Matched		
Variables	CRRT (*n* = 57)	CRRT + HA (*n* = 54)	*P*-value	CRRT (*n* = 38)	CRRT + HA (*n* = 38)	*P*-value
Age (years), mean ± SD	49 ± 21	47 ± 17	.54	47 ± 20	50 ± 16	.455
Males, *n* (%)	36 (63)	45 (83)	.017	31 (82)	30 (79)	.773
Use of vasoactive drugs, *n* (%)	40 (70)	24 (44)	.006	26 (68)	19 (50)	.102
Use of mechanical ventilation, *n* (%)	54 (95)	48 (89)	.435	37 (97)	35 (92)	.607
SOFA score, mean ± SD	14 ± 3	13 ± 3	.011	13 ± 3	13 ± 3	.59
APACHE II score, mean ± SD	32 ± 6	32 ± 5	.964	31 ± 5	33 ± 6	.156
McMahon score, mean ± SD	8 ± 3	9 ± 3	.278	8 ± 3	9 ± 3	.195
Pulse rate (beats/min), mean ± SD	102 ± 22	100 ± 24	.773	103 ± 23	100 ± 26	.564
Oximetry (%), median (IQR)	100 (98–100)	100 (99–100)	.338	99 (98–100)	100 (99–100)	.338
MAP (mmHg), mean ± SD	82 ± 16	86 ± 12	.133	83 ± 17	86 ± 13	.358
Causes of rhabdomyolysis, *n* (%)						
Sepsis	13 (23)	9 (17)		9 (24)	6 (16)	
Trauma	7 (12)	11 (20)		6 (16)	8 (21)	
Pancreatitis	18 (32)	14 (26)		3 (7)	4 (11)	
Other	19 (33)	20 (37)		20 (53)	20 (52)	
Pre-existing diseases, *n* (%)						
Diabetes	13 (23)	7 (14)	.177	9 (24)	7 (18)	.574
Hypertension	15 (26)	18 (33)	.419	13 (34)	15 (39)	.634
Neoplasms	4 (7)	3 (6)	1.00	2 (5)	1 (3)	1.0
Chronic pulmonary disease	5 (9)	1 (2)	.233	4 (11)	0 (0)	.123
AKI stage at the initiation of CRRT, *n* (%)						
AKI I	13 (22)	13 (24)	.542	6 (16)	10 (26)	.246
AKI II	22 (39)	24 (44)		14 (37)	14 (37)	
AKI III	22 (39)	17 (32)		18 (47)	14 (37)	
Peak CK (IU/l), median (IQR)	20 189(13 576–36 675)	35 694 (21 038–79 582)	<.001	19 478 (14 979–44 108)	36 206 (17 360–109 677)	.037
CK before CRRT (IU/l), median (IQR)	11 025 (4066–17 104)	21 484 (11 420–47 086)	<.001	11 982 (6961–19 773)	22 457 (11 919–50 297)	.005
Serum creatinine (μmol/l), median (IQR)	299 (194–471)	277 (177–402)	.21	224 (177–347)	174 (108–273)	.092
Urea nitrogen (mmol/l), median (IQR)	18 (11–28)	14 (10–21)	.133	16 (9–27)	16 (10–23)	.746
Albumin (g/l), mean ± SD	301 ± 6	32 ± 6	.197	30 ± 6	32 ± 6	.218
Globulin (g/l), mean ± SD	20 ± 6	21 ± 6	.89	21 ± 7	22 ± 5	.775
Haemoglobin (g/l), mean ± SD	102 ± 31	108 ± 34	.318	104 ± 30	110 ± 37	.437
Platelet count (×10^9^/l), median (IQR)	74 (40–137)	93 (48–128)	.596	76 (42–138)	108 (66–144)	.593
Leukocytes (×10^9^/l), median (IQR)	14 (9–22)	11 (9–19)	.217	13 (9–19)	11 (8–22)	.394
Calcium (mmol/l), mean ± SD	2.0 ± 0.26	2.0 ± 0.33	.992	2.0 ± 0.26	2.0 ± 0.33	.437
Phosphate (mmol/l), median (IQR)	1.7 (1.2–2.5)	1.4 (1.0–2.0)	.113	1.5 (0.8–2.1)	1.4 (1.0–2.0)	.668
Potassium (mmol/l), median (IQR)	4.6 (3.7–5.7)	4.3 (3.9–4.8)	.163	4.7 (3.7–5.3)	4.5 (4.1–5.1)	.414
CRRT treatment duration (h), median (IQR)	81 (38–173)	132 (60–290)	.009	94 (40–180)	142 (60–292)	.061
Time from initiation of CRRT to peak CK occurrence (h), median (IQR)	−17 (−36–6)	1 (−20–14)	.075	−16 (−35–9)	−4 (−20–18)	.275
Outcomes						
Duration of stay in ICU (days), median (IQR)	7 (3–13)	12 (5–24)	.009	8 (4–13)	13 (5–21)	.066
Length of hospitalization (days), median (IQR)	11 (5–24)	19 (9–39)	.012	11 (5–22)	19 (9–39)	.045
In-ICU mortality, *n* (%)	27 (47)	9 (17)	<.001	16 (42)	7 (18)	.025
In-hospital mortality, *n* (%)	27 (47)	10 (19)	<.001	16 (42)	8 (21)	.048
90-day mortality, *n* (%)	43 (75)	26 (48)	.002	26 (68)	18 (47)	.063

Before PSM, the CRRT + HA group exhibited a higher proportion of males (83% versus 63%, *P* = .017), higher CK before CRRT (21 484 versus 11 025 IU/l, *P* < .001), higher peak CK (35 694 versus 20 189 IU/l, *P* < .001) and longer duration of CRRT (132 versus 81 h, *P* = .009). In addition, the CRRT + HA group had lower SOFA scores (13 versus 14, *P* = .011) and a lower proportion of vasoactive drug utilization (44% versus 70%, *P* = .006).

After PSM, CK levels before CRRT (22 457 versus 11 982 IU/l, *P* = .005) and peak CK (36 206 versus 19 478 IU/l, *P* = .037) were significantly higher in the CRRT + HA group compared with the CRRT group. No differences were observed regarding other baseline variables between the two groups after PSM.

### Primary endpoints

**Figure 2:**
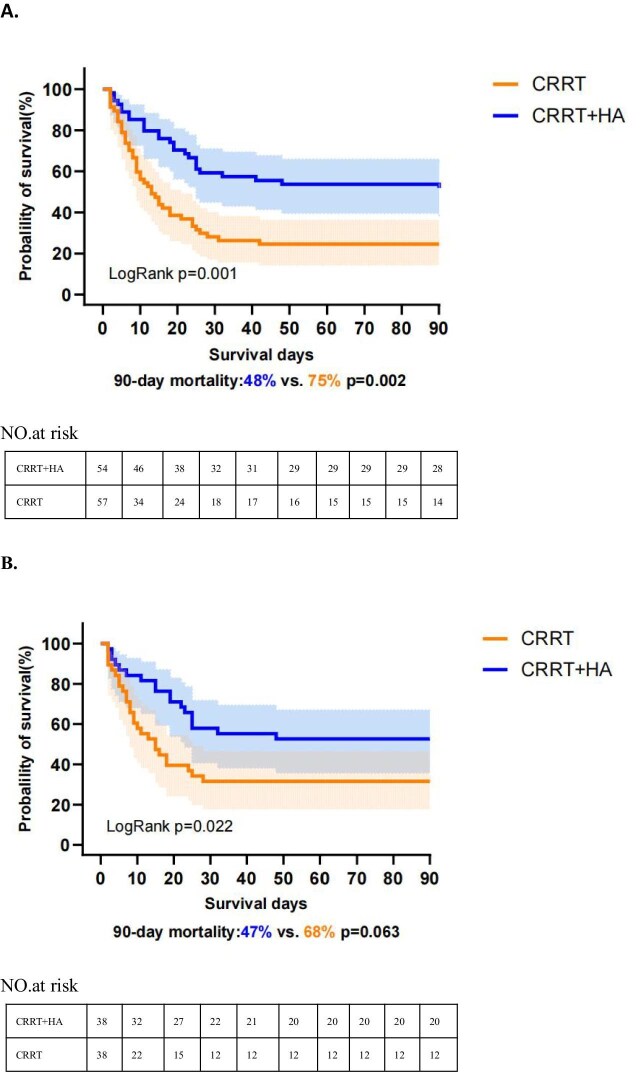
(A) Pre-matched and **(B)** post-matched Kaplan–Meier estimates of 90-day mortality.

As shown in Table 1, the 90-day mortality rate was significantly lower in the CRRT + HA group than in the CRRT group (48% versus 75%, *P* = .002) before PSM. Nevertheless, the difference in 90-day mortality between the CRRT + HA and CRRT groups after PSM was not significant (47% versus 68%, *P* = .063). However, the Kaplan–Meier survival curves showed a significant increase in the risk of death in the CRRT group at the 90-day follow-up between pre-PSM (logrank test, *P* = .001; Fig. 2A) and post-PSM (logrank test, *P* = .022; Fig. 2B). Meanwhile, patients in the CRRT + HA group exhibited lower ICU mortality between pre-PSM (17% versus 47%, *P* < .001) and post-PSM (18% versus 42%, *P* = .025), as well as lower in-hospital mortality between pre-PSM (19% versus 47%, *P* < .001) and post-PSM (21% versus 42%, *P* = .048; Table 1).

In contrast, the CRRT + HA group had a longer duration of stay in the ICU (12 versus 7 days, *P* = .009) and longer hospitalization (19 versus 11 days, *P* = .012) before PSM. However, patients in the CRRT + HA group after PSM experienced longer hospital stays (19 versus 11 days, *P* = .045), but there was no significant difference in ICU length of stay (Table 1).

The multivariate Cox regression analysis indicated that several factors independently contributed to 90-day mortality before PSM, including the need for mechanical ventilation support [HR 9.995 (95% CI 2.18–45.823), *P* = .003], elevated serum phosphate before CRRT [per 1 mmol/l; HR 1.461 (95% CI 1.149–1.858), *P* = .002] and higher McMahon scores at admission [per 1 score; HR 1.143 (95% CI 1.043–1.254), *P* = .004]. Moreover, the model showed that MAP [per 1 mmHg; HR 0.976 (95% CI 0.958–0.995), *P* = .012], duration of CRRT [per 1 h; HR 0.996 (95% CI 0.993–0.998), *P* < .001] and the CRRT + HA treatment model [HR 0.565 (95% CI 0.323–0.99), *P* = .046] were independent protective factors for 90-day mortality (Table 2). In the multivariate Cox regression analysis of 90-day mortality after PSM, similar results to those before PSM were obtained. Elevated serum phosphate before CRRT [per 1 mmol/l; HR 1.531 (95% CI 1.113–2.106), *P* = .009] and higher McMahon scores at admission [per 1 score; HR 1.15 (95% CI 1.006–1.313), *P* = .04] were identified as independent risk factors for 90-day mortality. Additionally, higher MAP [per 1 mmHg; HR 0.967 (95% CI 0.943–0.992), *P* = .009], longer duration of CRRT [per 1 h; HR 0.995 (95% CI 0.992–0.998), *P* = .002] and use of the CRRT + HA treatment model [HR 0.477 (95% CI 0.234–0.972), *P* = .042] remained independent protective factors for 90-day mortality (Table 2).

**Table 2:** Multivariate Cox regression analyses for 90-day mortality of RM and AKI patients before and after PSM.

	Unmatched multivariate		Matched multivariate	
Variables	HR (95% CI)	*P*-value	HR (95% CI)	*P*-value
Sex	1.013 (0.0.55–1.867)	.966	–	–
Age	0.998 (0.983–1.012)	.756	–	–
Use of vasoactive drugs	1.410 (0.708–2.809)	.328	2.139 (0.974–4.697)	.058
Use of mechanical ventilation	9.995 (2.18–45.823)	.003	2.577 (0.306–21.697)	.384
Modes of blood purification	0.565 (0.323–0.99)	.046	0.477 (0.234–0.972)	.042
MAP	0.976 (0.958–0.995)	.012	0.967 (0.943–0.992)	.009
CRRT treatment duration	0.996 (0.993–0.998)	<.001	0.995 (0.992–0.998)	.002
Phosphate	1.461 (1.149–1.858)	.002	1.531 (1.113–2.106)	.009
Globulin	0.963 (0.915–1.014)	.154	0.984 (0.924–1.048)	.614
McMahon score	1.143 (1.043–1.254)	.004	1.15 (1.006–1.313)	.040
SOFA score	1.034 (0.927–1.154)	.548	–	–
APACHE II score	0.997 (0.946–1.051)	.911	1.054 (0.972–1.142)	.207

### Secondary endpoints

**Figure 3:**
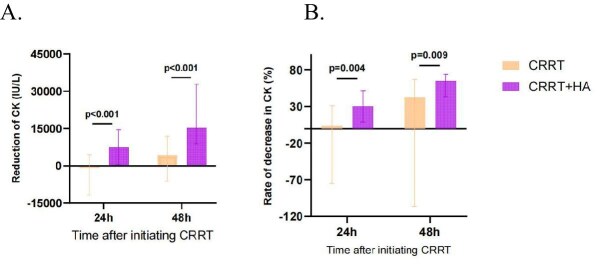
Efficiency of CRRT and CRRT + HA in the removal of CK at 24 and 48 h after RRT treatment. **(A)** The decrease in CK at each time point. **(B)** The decrease in rates of CK at each time point.

As shown in Fig. 3 and Supplementary File 1, after 24 and 48 h of CRRT, CK reduction and the rate of CK reduction showed consistent trends before and after matching, with more significant effects in the CRRT + HA group (*P* < .05).

We assessed the prognosis of kidney function in both groups of patients at the 90-day follow-up. At the 90-day follow-up, there was no significant difference in the recovery of renal function in the number of surviving patients between the two groups before PSM (*P* = .525) and after PSM (*P* = .639; Table 3 and Supplementary File 2). Specifically, after PSM, a total of 32 patients survived in both groups, with 20 in the CRRT + HA group and 12 in the CRRT group. In the CRRT + HA group, kidney function returned to normal in 19 patients (95.0%), while 1 patient (5%) developed CKD but did not require IHD. In contrast, in the CRRT group, the kidney function of 10 patients (84%) returned to normal, whereas 2 patients (16%) developed CKD but were not dependent on HD treatment (Table 3).

**Table 3:** The prognosis of kidney function after PSM.

Variables	CRRT (*n* = 38)	CRRT + HA (*n* = 38)	*P*-value
Complete recovery of kidney function, *n* (%)	10 (84)	19 (95)	.639
Partial recovery of kidney function, *n* (%)	2 (16)	1 (5)	
Dialysis dependent, *n* (%)	0 (0)	0 (0)	
Renal function recovery time (days), mean ± SD	27 ± 18	21 ± 16	.378
Time of onset of polyuria (days), median (IQR)	11 (4–15)	10 (6–21)	.720
Urine output during polyuria (ml/day), median (IQR)	3155 (2670–3564)	3001 (2629–3462)	.596

**Figure 4:**
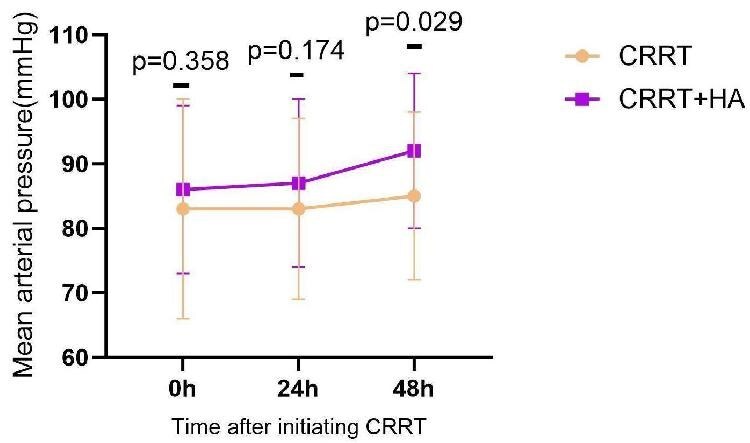
Changes in MAP in patients at the start of treatment and 24 and 48 h after CRRT or CRRT + HA treatment.

In addition, patients in the CRRT + HA group had a higher MAP 48 h after treatment (92 ± 12 versus 85 ± 13 mmHg, *P* = .029, respectively), consistent with the pre-PSM results (Fig. 4 and Supplementary File 5). In contrast, there was no statistically significant difference in the change in vasoactive-inotropic scores at 24 and 48 h after treatment between the two groups (*P* > .05; Supplementary File 6).

### Safety endpoints

**Figure 5:**
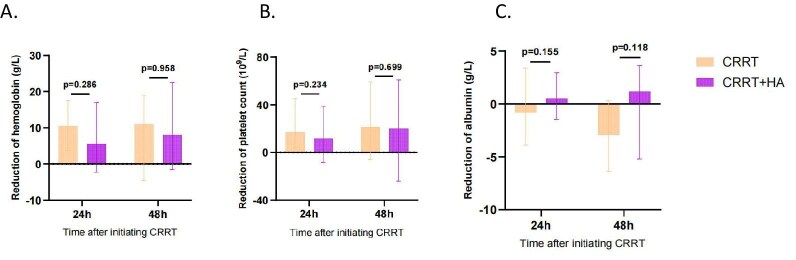
The reduction of haemoglobin, platelets and albumin at 24 and 48 h after CRRT treatment in the CRRT and CRRT + HA groups. **(A)** The median reduction of haemoglobin at each time point. **(B)** The median change in the reduction of platelets. **(C)** The median change in the reduction of albumin.

Before (Supplementary File 3) and after (Fig. 5) PSM, no significant differences were detected in the levels of albumin, platelets and haemoglobin lost (*P* > .05) between the two groups after 24 and 48 h of CRRT.

There was no statistically significant difference in circuit clotting events between the two groups (*P* = .477). However, hypotensive events were significantly prevalent among patients in the CRRT group (39.5% versus 20.2%, *P* = .002; Table 4). In the CRRT and CRRT + HA groups, pre-PSM circuit clotting events (*P* = .949) and hypotensive events (42.6% versus 18.1%, *P* < .001) were consistent with post-PSM events (Supplementary File 4).

**Table 4:** Adverse events after PSM.

Variables	CRRT (*n* = 38)	CRRT + HA (*n* = 38)	*P*-value
CRRT cases, *n*	119	109	
Coagulation during CRRT, *n* (%)	31 (26.1)	24 (22.2)	.477
Filter coagulation, *n* (%)	31 (26.1)	21 (19.3)	
Venous pot coagulation, *n* (%)	1 (0.84)	3 (2.8)	
Adsorbent cartridge coagulation, *n* (%)	–	9 (8.3)	
Hypotension, *n* (%)	47 (39.5)	22 (20.2)	.002
Hypotension during haemoperfusion, *n* (%)	–	15 (13.8)	

In addition, we found that patients in the CRRT + HA group demonstrated a significant decrease in platelet and haemoglobin levels after each HA. In contrast, albumin levels did not show significant changes before and after HA (Table 5).

**Table 5:** Haemoglobin, platelets and albumin consumed per HA.

Variables	Before HA treatment	After HA treatment	*P*-value
Platelet count (×10^9^/l), median (IQR)	78 (45–119)	67 (38–115)	.009
Haemoglobin (g/l), median (IQR)	91 (76–112)	87 (76–104)	.003
Albumin (g/l), mean ± SD	31.6 ± 5.4	31.3 ± 6.0	.689

## DISCUSSION

This retrospective study involved 111 patients with RM and AKI, aiming to explore the efficacy and safety of CRRT + HA in treating patients with RM and AKI. We found that with the same treatment mode and therapeutic dose of CRRT (20–25 ml/kg/h), the combination therapy reduced mortality in patients with RM combined with AKI and effectively decreased CK levels without increasing the incidence of adverse events. RM is a clinical syndrome characterized by the breakdown and necrosis of skeletal muscle tissue and the release of its cellular contents into the circulation due to various causes [1–5]. Myoglobin, with a molecular weight of 17 kDa, is the primary pathogenic substance causing AKI in RM patients [32, 33]. Myoglobin induces AKI mainly by inducing renal vasoconstriction, tubular obstruction and tubular oxidative damage [1, 11, 13, 34–36]. Normally functioning kidneys are capable of removing myoglobin effectively and quickly. However, when kidney function is impaired, the myoglobin removal capacity decreases. Here, our research aimed to explore efficient *in vitro* methods for myoglobin removal.

In our study, the peak CK and initial CK values in the CRRT + HA group were significantly higher compared with those in the CRRT group both before and after PSM. This observation may be because of the selective use of HA therapy in our institution, which is primarily reserved for patients with markedly elevated CK levels. Assanangkornchai *et al.* [37] found that an increased rate of >1000 U/l/h was significantly associated with AKI. Joanna *et al.* [38] also found that peak CK of at least 5000 IU/l is 55% specific and 83% sensitive for the prediction of AKI requiring RRT. However, a retrospective study involving 161 patients revealed serum myoglobin as a superior predictor of AKI and 90-day mortality versus CK among patients experiencing RM after exertional heatstroke. Moreover, CK was not a risk factor for 90-day mortality [39]. Therefore the implications regarding CK levels in patients with RM need to be explored further.

Recent studies have demonstrated that CRRT + HA can effectively remove myoglobin and CK [22, 40]. A recent prospective study demonstrated that CytoSorb adsorbent (CytoSorbents, East Princeton, NJ, USA) was effective in clearing myoglobin, with CK clearance being statistically significant only at 10 min (*P* = .005) and 1 h (*P* < .001) after treatment. The median relative rates of change in CK at the specified time points (10 min, 1 h, 3 h, 6 h and 12 h after initiation of treatment) were −14.3%, −2.8%, 0.1%, 1.0% and 0.0%, respectively [41]. Another small study showed that myoglobin concentration decreased more rapidly when CytoSorb was added to CVVHD and that the CytoSorb + CVVHD group cleared CK, but the clearance time was shorter, with a CK clearance of <5% at ≈2 h, compared with near-zero clearance by CVVHD using a high cut-off haemofilter [42]. The above studies are consistent with the results of our study that HA can clear some CK, but for a shorter duration. In our study, in contrast, the significant decrease in CK at 24 and 48 h after treatment was partly due to the fact that we would have changed the adsorption device once after 10 h of HA treatment if the CK in the CRRT + HA group had not been <10 000 U/l, and partly due to the fact that we had a longer observation period, so we took into account that the patient's primary diseases were already under control to a certain extent. Unfortunately, our ability to compare the degree of myoglobin reduction and clearance efficiency between the two groups was limited. This constraint arose from the use of ElectroChemiLuminescence (Roche, Rotkruez, Switzerland), which can only provide relative results when myoglobin concentrations are >3000 ng/ml.

In a study that included 43 patients with RM and AKI, treatment using CRRT together with Cytosorb for >90 min reduced the in-hospital mortality rate of patients from the predicted 92.5% to 67.4% [22]. These findings parallel those from our study, which revealed a reduced mortality rate after CRRT + HA for RM and AKI. Different from this research, our study provides a more accurate comparison by directly assessing the mortality rate between the CRRT + HA group and the CRRT group. However, our study is limited by the baseline imbalance between the two groups, which may bring about bias in mortality outcomes. To mitigate this issue, we further compared 90-day mortality between the CRRT + HA and CRRT groups after reducing the baseline imbalance using PSM. After all these efforts, we still concluded that patients in the CRRT + HA group had a lower 90-day mortality rate; but unfortunately, this disparity did not achieve statistical significance. The observed outcome is likely attributed to the reduced sample size after PSM. Therefore, high-quality prospective studies and trials are warranted to validate these results. Continued exploration of the duration and intensity of HA treatment remains imperative.

Previous studies have shown that the need for mechanical ventilation in patients undergoing RRT is an independent risk factor for death [5, 43]. In our study, we found that the need for mechanical ventilation was indeed an additional risk factor for death before PSM, but this risk decreased after matching. This shift may be due to a selection bias caused by the exclusion of certain mechanically ventilated patients after PSM. Additionally, our study revealed that the McMahon score and serum phosphate concentration before initiation of therapy were independent risk factors for 90-day mortality. A previous report demonstrated that mortality among patients with a McMahon score >10 was 61.2%, which decreased to 2.3% for those with a McMahon score <5 [38], in line with our research results. The Critical Care Committee of the American Association for Surgery also acknowledges the McMahon score as a prospectively validated risk assessment tool for identifying patients at higher risks, as stated in their 2022 consensus document [44]. Hyperphosphataemia occurs due to the release of phosphate from damaged muscle cells. Usually, early hyperphosphataemia decreases as phosphate is eliminated from the urine [44]. Therefore, significant hyperphosphataemia in a patient may suggest severe muscle tissue damage and notably impaired kidney function. A study of blood phosphorus and child mortality has showed that hyperphosphataemia before and during CRRT predicts a higher 90-day mortality rate [45]. Hyperphosphataemia was also shown to portend higher mortality in a study by Thongprayoon *et al.* [46].

In our study, higher MAP [per 1 mmHg; HR 0.967 (95% CI 0.943–0.992), *P* = .009], longer duration of CRRT [per 1 h; HR 0.995 (95% CI 0.992–0.998), *P* = .002] and adoption of the CRRT + HA treatment model [HR 0.477 (95% CI 0.234–0.972), *P* = .042] were found to be protective factors for 90-day mortality. The MAP level serves as an indicator of patient condition, thus naturally rendering it a protective factor. In our study, we observed that a longer duration of CRRT treatment serves as a protective factor for mortality. This may be related to the higher peak CK in our selected patients, which was 19 478 IU/l and 36 206 IU/l in the CRRT and CRRT + HA groups, respectively. Assanangkornchai *et al.* [37] found that a peak value of serum CK >10 000 IU/l or >16 000 IU/l increased the probability of AKI and RRT. So the need for CRRT therapy may be greater in our selected patients. It has also been shown that patients treated with CRRT have a more favourable recovery of kidney function and a lower mortality rate than patients treated with IHD [47].

In 1964, Yatzidis [48] pioneered the use of activated charcoal to treat renal diseases, marking the inception of HA as a treatment modality. However, early adsorbents faced challenges such as thrombus formation due to dislodgement and exhibited poor biocompatibility, leading to complications like allergy, hypotension, severe platelet destruction and notable albumin loss. Consequently, these initial HA techniques did not see widespread adoption. As technology has advanced, new adsorbents have displayed better biocompatibility and safety, reducing adverse events [25]. It was found that haemoadsorption devices like the HA series (including HA130, HA230, HA330/380; Jafron, Zhuhai, China) have led to only a few instances of thrombocytopaenia in the treatment of sepsis, which were transient and considered not clinically significant [49]. Montin *et al.* [50] demonstrated that HA does not adsorb beneficial substances such as albumin and causes unnecessary losses. This is consistent with our findings that there was no statistical difference in haemoglobin, platelets and albumin depletion between CRRT + HA and CRRT after 24 h and 48 h of treatment. In addition, we also noticed a decrease in the platelet count by ≈11 × 10^9^/l and haemoglobin by ≈4 g/l following each HA session in the CRRT + HA group, whereas albumin levels remained relatively stable before and after HA sessions.

Surprisingly, more hypotensive events occurred during treatment in patients in the CRRT group than in the CRRT + HA group. This disparity may be attributed to several factors: patients in the CRRT group were hospitalized mainly due to conditions such as infection and pancreatitis; and HA380, a component of the CRRT + HA protocol, demonstrated effectiveness in reducing the need for vasopressors, thereby aiding in maintaining the haemodynamic stability of patients. Träger *et al.* [51] discovered that HA therapy reduces the concentration of inflammatory mediators (interleukin-6, interleukin-10) and stabilizes and restores haemodynamics. Similarly, in another study of patients with septic shock requiring RRT, HA was found to approximately halve catecholamine requirements within 24 h [52]. And the above study is consistent with our finding that HA is more favourable for regaining haemodynamic stability.

Our current study has several limitations. First, this is a single-centre study with a limited sample size, and the patients included were limited to those in the ICU, which may not fully represent all patients with RM and AKI. A multicentre study should be conducted to validate our findings across a broader patient population. Second, our study was retrospective, making the effective control of baseline variables challenging. Third, limited by our institution's myoglobin testing technology, we were unable to obtain the comparative efficacy of the two blood purification modalities in terms of myoglobin reduction and clearance efficiency. Finally, although we reported a large cohort of RM patients treated with CRRT, the overall sample size was relatively small. Consequently, more extensive randomized controlled studies are needed to obtain more robust evidence in the future.

## CONCLUSION

Our study showed that CRRT + HA is associated with reduced mortality in patients with RM and AKI, without inducing severe clinical adverse events, thus CRRT combined with HA may be a better treatment modality for patients with RM and AKI. However, large sample randomized controlled trials are still needed to corroborate these findings and provide further insights into its myoglobin clearance efficiency.

## Supplementary Material

sfae406_Supplemental_File

## Data Availability

The data underlying this article are available in the article and in its online supplementary material.
