# Supplementary material for: Efficacy of haemoadsorption combined with continuous renal replacement therapy in patients with rhabdomyolysis and acute kidney injury: a retrospective study
Source: Clin Kidney J. 2024 Dec 17;18(2):sfae406. doi: 10.1093/ckj/sfae406 (PMC11803309; doi:10.1093/ckj/sfae406)
Supplement: sfae406_Supplemental_File [file sfae406_supplemental_file.pdf]

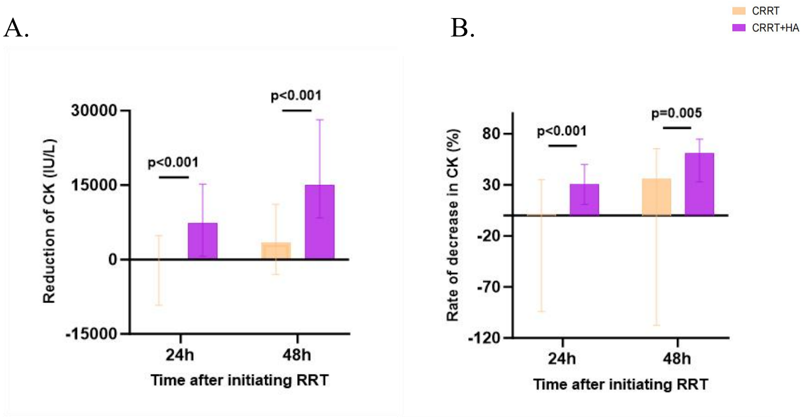

Additional file 1. Efficiency of CRRT and CRRT+HA in the removal of creatine kinase at 24, and 48 hours after RRT treatment in the CRRT and CRRT+HA groups before PSM. Panel (A) shows the decrease values at each time point, and Panel (B) shows the decrease rates of creatine kinase at each time point.

Additional file 2. The prognosis of kidney function before PSM.

Variables	Control arm CRRT, n = 57	Intervention arm CRRT+HA, n =54	p-value
Complete recovery of kidney function (n%)	12 (85.7%)	27 (96.4%)	0.525
Partial recovery of kidney function (n%)	2 (14.3%)	1 (3.6%)	
Dialysis-dependent (n%)	0 (0%)	0 (0%)	
Renal function recovery time (days)	33 ± 17	19 ± 14	0.025
Time of onset of polyuria (days)	13 (8, 18)	9 (5, 17)	0.558
Urine output during polyuria (ml/day)	3178 (2811, 3562)	3100 (2625, 3415)	0.373

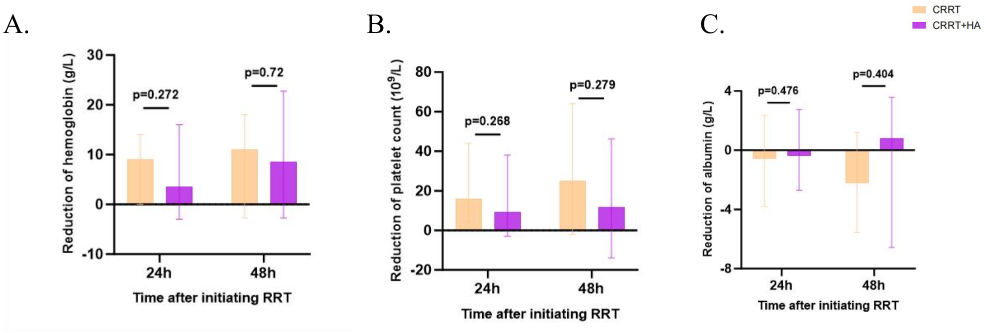

Additional file 3. The Reduction of hemoglobin, platelet, and albumin at 24, and 48 hours after RRT treatment in the CRRT and CRRT+HA groups before PSM. Panel (A) shows the median Reduction of hemoglobin at each time point, Panel (B) shows the median change reduction of platelet, and Panel (C) shows the median change reduction of albumin.

Additional file 4. Adverse events before PSM.

Variables	CRRT group,n=57	CRRT+HA group,n=54	p-value
Coagulation during CRRT(n%)	42（24.9%）	39（25.2%）	0.949
Hypotension during CRRT(n%)	72（42.6%）	28（18.1%）	＜0.001

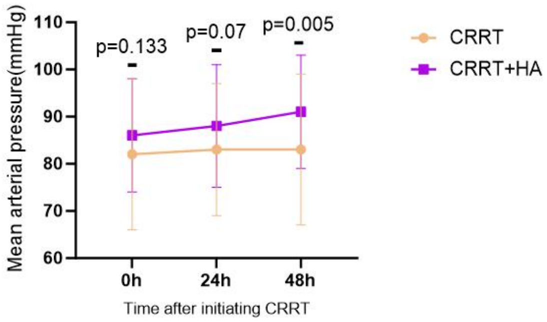

Additional file 5. Changes in mean arterial pressure in patients at the start of treatment, 24 hours and 48 hours after CRRT or CRRT+HA treatment.

A.

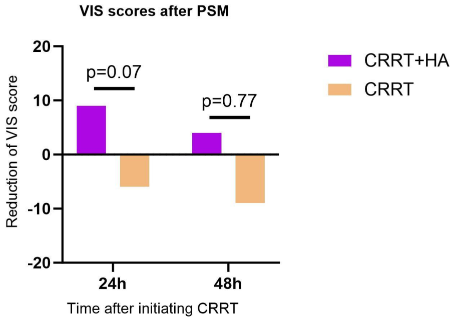

B.

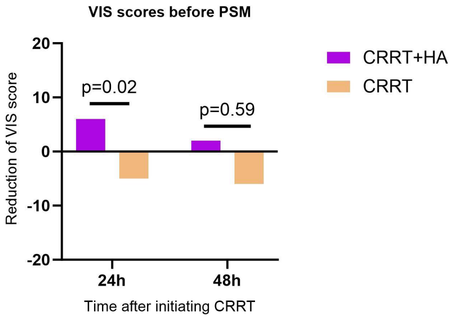

Additional file 6. Change in VIS scores before and after PSM. Panel (A) VIS scores after PSM, Panel (B) VIS scores before PSM.
